# Is Affectivity Passive or Active?

**DOI:** 10.1007/s11406-017-9926-9

**Published:** 2017-12-05

**Authors:** Robert Zaborowski

**Affiliations:** Institute of Philosophy, University of Warmia and Mazury, Olsztyn, Poland

**Keywords:** Emotion, Feeling, Affectivity, Passivity, Activity, Heterogeneity of affectivity

## Abstract

In this paper I adopt Aquinas’ explanation of passivity and activity by means of acts remaining in the agent and acts passing over into external matter. I use it to propose a divide between immanent-type and transcendent-type acts. I then touch upon a grammatical distinction between three kinds of verbs. To argue for the activity and passivity of affectivity I refer to the group that includes acts of transcendent-type and whose verbs in both voices possess affective meaning. In the end I focus on cases in which an act of affective *f*-ing is mirrored in its object as being affectively *f*-ed.

## Introductory Remarks

Let me start with three provisos.

First, I want to underline the asymmetry of the active/passive distinction and action/passion distinction. This is for the most part a linguistic issue: we tend today to link activity with action more than we link passivity with passion. For example, being cooked is a passive state but it is not a passion as we understand this word. Therefore, while action and activity cover more or less the same field, passion is much narrower than passivity.

Second, passivity is often identified with reactivity. Seen from this angle, thought and emotions^1^ are doubtlessly different. For I can very well ponder a problem out of nothing, whereas it is hard, or even impossible, to feel out of nothing. Examples of the inability to produce a usual feeling when it is absent, e.g. when contemplating my favourite painting, listening to a piece of music, drinking my preferred wine, are patent evidence of this: I know or feel that I am used to feeling such and such in some circumstances, yet when my usual feeling does not emerge on a particular occasion there is nothing I can do about it. Sometimes, even worse, it happens when I am back in a place or circumstance in which I felt such and such, and this time this feeling is absent. I then realise that I miss it, and want to feel it, but do not succeed in producing or bringing it back. This is also valid for unpleasant things: at some point I am afraid of this or that or overwhelmed by a sad mood or thoughts, and at another time a very similar - or even identical - environment does not affect me at all.^2^ Such accidental changes of heart can be systematized in a more general life approach and take a form detailed by Thomas Mann in his early novel *Disillusionment*: “the great and general disappointment which everything, all of life, has in store” (Mann 1936). Here the interlocutor is still reflecting, but for years has been left unaffected by any event he thinks could or should affect him. If what I say is correct, this would speak against cognitive approaches to affectivity, especially its strong version, which claims that all affective phenomena are inextricably based on cognition - unless it can be proved that the two situations, one in which I am feeling and another in which I remain indifferent to what has otherwise affected me, are cognitively different. On the other hand, this situation shows the richness and variety of affectivity insofar as some genera, or maybe species of feeling, say of anger, can be produced or more easily produced, while others cannot.

To go further: we speak about practical thought and abstract thought, where the latter seems not to be reactive in any sense whatsoever. For instance, I can begin to undertake mathematical operations without their being necessarily reactive to some stimulus. But what would a distinction between practical and abstract feelings mean? It seems that in the realm of affectivity such a distinction doesn’t make sense. Consequently, thought does not have to be reactive while feeling is a reaction or, if you prefer less biological and more philosophical language, a response to something that occurs, say, something that supervenes on a modification.^3^ In this sense feelings are reactive/responsive, because they are secondary to something else. Accordingly, if passivity is tantamount to being reactive/responsive, then obviously feelings, unlike thoughts, can be said to be passive. If, however, affectivity is understood as or identified with spontaneity (as above, when feeling is read as not being produced at will) we arrive at a strange result, at first glance at least: the same phenomenon is both passive and spontaneous. Read this way it looks curious. If we replace passive with responsive, as I have just done, we see that what overlaps here is not passivity and spontaneity but spontaneity and responsiveness. On the one hand a feeling is a response, and on the other hand the nature of this response is such that it is spontaneous (as opposed to a deliberated or calculated response). In consequence, passivity qua passivity is broader than passivity qua responsiveness. Hence, if it may be said that affectivity is passive without further qualification, this is only insofar as it is meant to be a response.

Third and finally, if the activity/passivity distinction is understood in terms of acting upon/being acted upon, this means that for a determinist, especially a hard one, one who claims that any event or act is produced by other events or acts, no event or act is active apart from - theoretically - the first of the chain of events or acts. Therefore, if one is a consistent determinist, not only feeling but all other mental acts or states are passive. It is not only what is felt, but also what is thought that is the result of a correlation of different factors, say the chemistry of the brain, which in turn is a result of our being no more and no less than, to use the common phrase, molecular machines working as sophisticated clocks.^4^ If for a determinist there is any difference between feelings (traditionally taken to be passive) and thoughts (traditionally taken to be active), this difference may be only of degree.

Therefore, if I want to speak about the activity and passivity of mental acts, and especially about the activity and passivity of affectivity, through the simple act of uttering such concepts I reject determinism and declare myself a non-determinist. While determinism rules out activity in a strong sense - that is, something’s being active without being passive, since any event in the chain of events is produced by a preceding one and produces a succeeding one (and only in this sense can it be seen as active), non-determinism doesn’t rule out passivity. For this reason it is worth considering here. Let us then consider non-determinism for a moment.^5^

## A Quick Historical Overview^6^

I have been applying concepts such as activity and passivity to the realm of affectivity and, thereby, have not been presenting myself as a consistent determinist. But who am I apart from being a non-determinist? It seems as if I accept that there are some affective events or acts that are not produced by other events or acts, be they affective or not affective. But what does this mean?

Let me start with examples belonging to a more transparent domain. When it comes to physical acts, activity is opposed to passivity as cutting is opposed to being cut or burning to being burnt. Such typical examples are frequently borrowed from Aristotle’s *Categories*,^7^ but, often Aristotle’s examples are similar or the same as those given in Plato:

“[...] take another point: if a man does anything, must there be something which is also acted upon by this doer of the thing? [...] And does it suffer what the doer does, and is the effect such as the agent’s action makes it? I mean, for example, when one strikes a blow something must needs be struck? [...] And if the striker strikes hard or quick, the thing struck is struck in the same way? [...] Hence the effect in the thing struck is such as the striker makes it? [...] And so again, if one burns, something must be burnt? [...] And again, if one cuts, the same may be said? For something is cut. [...] And if the cut is large or deep or sore, the cut made in the thing cut is such as the cutter cuts it?” (transl. W. R. M. Lamb^8^).

Plato’s formulation is even more attractive in this context since he not only gives examples of both categories, which are the same as in Aristotle, but he also takes into account in a clear and explicit way the quality of one category common to the second category: *οἷον* ἂν ποιῇ τὸ ποιοῦν, *τοιοῦτον* τὸ πάσχον πάσχειν (476d3–4, transl. W. R. M. Lamb: “the patient receives an effect of the same kind as the agent’s action”).

Commonly and traditionally, affectivity is considered to belong to the province of passivity, and as such is opposed to activity. For instance for Aquinas,

“[p]assion is the effect of the agent on the patient” (transl. The Fathers of the English Dominican Province^9^).

In a similar vein, according to Descartes

“[...] the term ‘passion’ can be applied in general to all the thoughts that are thus aroused [= ‘that are aroused by cerebral impressions’] in the soul without the concurrence of its will, and therefore without any action of the soul itself; for whatever is not an action is a passion.” (transl. J. Bennett (2010)^10^)

Accordingly,

“when the soul uses its will to make itself have some thought that is not just intelligible but also imaginable, this thought makes a new impression in the brain; this is not a passion within the soul, but an action - and it is what is properly called ‘imagination’” (transl. J. Bennett (2010)^11^).

In all cases, however, the opposition seems to rule out the middle term - to cut oneself - because what is doing the cutting is different from what is cut, and vice versa. Those who allude to a case of a man who cuts himself and say that a man cuts himself speak too vaguely. To be more accurate they should speak about someone who cuts one of his hands with his other hand, etc.^12^

## Preliminary Distinction

In general, active versus passive (or activity versus passivity) is about doing versus being done. But in what sense? Anthony Kenny pictures the difference between activity and passivity differently when it is related to the physical (i.e. non-psychological) and the mental (i.e. psychological) realms. He writes:

“If Peter has painted his house, then Peter’s house must now be different from what it was before he painted it; but if Peter has looked at his house, it may now be exactly the same as it was before he looked at it.” (1963, 196)

And it is much the same for many others cases: if I push someone, she is pushed and this being pushed is a modification that happens to her without her acting.^13^ Instead, if I think about someone, her being thought about by me does not affect the person thought about by me. To use Aquinas’ expression (Kenny’s translation), painting something and pushing someone “pass over into external matter”, whereas looking at something and thinking about someone “remain in the agent”.^14^ Consequently, I call the former a transcendent-type (T-type) of act and the latter an immanent-type (I-type) of act. The distinction is visible if, for example, the person I think about is told that I think about her. This can then bring about a modification in her, yet this happens by means of being told that she is being thought about by me and not by simply being thought about by me. In such a case it should be inquired how much modification results from being thought about and how much results from being told about being thought about. Telling resembles activities such as painting and pushing, while thinking about resembles looking at.^15^ Now imagine that the person I think about is not told about my thinking about her but for some reason (that probably should be better specified) she imagines, supposes, or thinks that I think about her. What then? Or to make the contrast even more visible: some person for some reason imagines, supposes, or thinks that I think about her whereas, in fact, I do not. Is there any difference in her imagining, supposing, or thinking that I think about her in cases where I do or do not think about her? I don’t think so, except if a kind of perceptual empathy allowing her to distinguish whether she is thought about or not is admitted.

This is why, if a modification of a person I think about occurs, it does not result from my thinking about her (*unless* she is told that she is thought about by me, but then the modification comes from being told and not from being thought about), but from her imagining, supposing, or thinking about what I (supposedly) do. This conclusion leads, then, to a distinction between three cases with respect to activity and passivity:

- thinking about someone without her being told about it,

or:

- thinking about someone followed by her being told about it,

and, as for the person being thought about:

- her thinking about my thinking about her, with or without being told about it.

The first case brings no modification,^16^ whereas the second and the third do. On the other hand, the first and the third act are of I-type while the second act, because of the second segment of this complex act, is of T-type. I-type brings out a modification but only an *immanent* modification - this is a modification in relation to the agent. A modification in relation to the object, which for this reason can be called a *transcendent* modification, occurs only in T-type acts, that is, in acts in which an agent and an object are involved and are joined by this T-type act. That said, I pass on to my central question, which is: to what category does affectivity belong, to passivity or activity? In what follows I shall show that affectivity, though it is mental like thinking, is in some cases a T-type act, like painting. And if affectivity is a T-type act, in such cases this entails both its activity and passivity.

## Grammatical Distinction

Affectivity is often considered to be passive. In his influential paper “The Passivity of Emotions” R. M. Gordon builds his argument on the grammatical distinction of voices.^17^ Thus he gives the following examples: “amused, annoyed, astonished, delighted, depressed, embarrassed, frightened, horrified, irritated, miffed, overjoyed, pleased, terrified, surprised, troubled, upset, and vexed”, plus some “participial ancestors”, e.g. “afraid and sad”. He takes them to support “grammatical evidence” of the fact “[t]hat the so-called “emotions” belong to the category of “passions”” simply because “the great majority of adjectives designating emotions are derived from participles” (Gordon 1986, 373–374).

But it is easy to see that, characteristically, all Gordon’s examples are biased. They are forms of verbs that do not refer to emotions because in their active voice they are causative.^18^ *To amuse*, *to annoy*, *to astonish* etc. have nothing affective in themselves, but only their result. I would rather say that even if I amuse/annoy/astonish/etc. you, with the intention of amusing/annoying/astonishing/etc. you, it is rather a posteriori that my intention can be ascribed an affective meaning (only) insofar as I am successful in my amusing/annoying/astonishing/etc. you, and the result occasioned is your being amused/annoyed/astonished/etc. But it is not sine qua non and you may well be amused/annoyed/astonished/etc. without my intention of amusing/annoying/astonishing/etc. you, e.g. by my saying something or by means of a gesture. If so, this kind of verb has something to do with affectivity, but attains it only contingently. The concept of my amusing/annoying/astonishing/etc. you is added to what I have done. There is no such thing as amusing/annoying/astonishing/etc. *in concreto*, but only by doing something that may or may not amuse/annoy/astonish/etc. This is an abstraction built onto the whole of what is produced as amusement, that is, someone’s being amused.

But it is also easy to see that this is only one category of verbs. And if Gordon’s examples are one-sided, then his conclusion, if only grammatically, begs the question. Selecting verbs of which the past participles refer to passive affective acts in order to argue for the passivity and against the activity of affectivity is defective, especially because we can consider other kinds of verbs that stand beyond “the “great majority” of emotion adjectives” Gordon (1986, 374, n. 5) speaks about.^19^

I think that apart from the above group (= 1), there are still two other kinds of verbs to be considered. One (= 2) is composed of verbs referring to affectivity in their active voice. As a matter of fact, there are two subgroups here: one (= 2a), of which there is no intelligible passive voice (e.g. rejoice), and the other (= 2b), of which the passive voice can be and is constructed, but its sense is affectively irrelevant (e.g. miss). Another group (= 3) is of verbs for which both the active and passive voices refer to affective meaning (e.g. love).

As for group 1, the one exploited by Gordon, its active voice has no mark of affectivity. What passes over into the subject being *f*-ed is not a feeling of *f*. This is an act which may in special circumstances be regarded as affective, but may not be paired with the intention of producing a feeling of being *f*-ed and, even if it is, it is not the act itself but its intention that is referred to. Accordingly, it may not be affective, and if it appears to be so, this is because of factors other than the act itself, the act of *f*.

As for group 2, both its subgroups, 2a and 2b, look active, at least grammatically. Let us consider them separately.

Group 2a looks active. But there is nothing passing over into the external domain or, to use the alternative formulation, all of it remains in the agent, because there is no existent object at all. This is because - this is the core feature of this subgroup - the affective act is contained in the feeling subject. For instance, when I go to opera and this makes me *fear*, *rejoice*, *enjoy*, *sorrow*, *grieve*, *regret* going to opera there is nothing of my joy, sorrow, grief or regret that passes over into external matter.^20^

In what concerns 2b, this subgroup includes verbs where the passive voice is intelligible (e.g. *miss*/*missed*, *hope*/*hoped*). It does not, however, describe the affective situation of the object of *f* but pertains to its relation to the subject of *f* without any affective meaning, e.g. that such a person is the one another misses. This says nothing about her affectivity being such and such, let alone modifies her affectively.

Although 2a and 2b are grammatically similar (no passive voice affectivity-related), they differ in terms of the absence of their objects: in 2a the object is virtually non-existent, while in 2b it is absent physically.^21^ One may also remark that 2b is in a sense the opposite of 1 since, for both of them, of their two voices only one (passive for 1 and active for 2b) is affectivity-related, while the other (active for 1 and passive for 2b) is affectivity-unrelated. A further analogy between 2b and 1 is that the affectivity-unrelated voice may be filled with affective meaning but this is only if additional requirements are satisfied, not per se*.* 22

Although I could be satisfied enough with groups 2a and 2b insofar as the acts they include are affective and active, I wish to focus on group 3. This is because, since I have accepted Aquinas’ distinction between active and passive by means of *passing over into external matter* versus *remaining in the agent*, I am committed to looking for a stronger example of affectivity than the I-type, even if it is active. I want it to be of the same kind as painting, that is, where the subject modifies the object by *f* in such a way that the latter is *f*-ed, and where both *f* and being *f*-ed are affective. Then, I believe, any ambiguity as to the activity and passivity of affectivity will be removed. For that I need an example of feeling which is of T-type. This is the case for group 3. It includes acts of T-type and, moreover, active and passive voices of relevant verbs are loaded with affective meaning. To this group belong love,^23^ admiration, hatred,^24^ envy, contempt, honour, and abhorrence.^25^

## Final Evidence

Let us then look at loving versus being loved. While it seems rather uncontroversial that *being loved* belongs to I-type, for it *remains in the agent,* 26 *loving* another person^27^ is or is supposed to be a T-type act, that is, it *passes over into external matter*, in this case *over into* another person.

Surprisingly, as soon as I accept that love is of this kind - and the same is valid for other items of this group - an objection appears. I have just suggested that loving is similar to painting and dissimilar to thinking to the extent that it passes over into the object of love. But it is easy to notice that while you cannot paint a house without the house being painted, one can love a person who does not feel loved. What is more, this is maybe what commonly happens. Consequently, unambiguous and convincing evidences of T-type, that is of affective acts *passing over into another matter*, turn out to be less numerous than the elements of group 3.

Answering this objection is not difficult. First and foremost I am not investigating the nature of love and other elements of group 3. Neither I am asking if and why they are of such a character as to pass over another person. It seems to me that it would be odd to admit that when loving is mirrored by being loved,^28^ loving is active, and when it is not mirrored, it is passive. Loving and other elements of group 3 and group 2 are active anyway. But since I am looking for strong and unambiguous evidence I refer to an example of *loving* being mirrored by *being loved* because, in this case, given the passivity of being loved, loving has to be considered active so that proof of the activity of affectivity is provided, and with it its passivity.

Let then us recognize that loving is *not always* followed by the feeling of being loved. If it is not, this must be accepted as a sad or unfortunate accident. This is because love is typically conceived as not limited to being contained in the subject, but also, or mainly, as intended to pass over into the object. Love that doesn’t reach the object of love is considered uncompleted.^29^ The idea of love as intended not to pass over into its object and to remain in the subject would seem eccentric or even absurd, and someone who would undertake, if I may say so, to love with intention of her love never passing over into the object of her love even to the slightest extent would be taken for a fool. Consequently, even if love does not always successfully pass over into its object and does not reach it, this is not what is expected nor believed to be its completion. Let me then acknowledge that love is an example of a T-type act because it inherently contains the intention of acting upon the beloved. What is crucial to my argument is that it *happens* to pass over into the beloved.^30^

## R. G. Collingwood’s View

There is, however, a quite different approach to the question of the activity and passivity of affectivity. In his last work, published a few months before his death, R. G. Collingwood attacked the issue directly:

“5.4. *Is feeling active or passive?* Once more I do not know, and here I cannot give even a methodological answer. [...]. 5.43. [...] The question whether when I feel I am doing something or having something done to me is a question that cannot be settled by appeal to argument [...]. 5.44. The only way of settling it is by appeal to reflection upon the immediate, unphilosophical, unargumentative consciousness of feeling. The question is: ‘How does feeling present itself to a man immediately conscious of it? A something he does or as something done to him?’ 5.45. The immediate consciousness of feeling will not answer this question [...]” (1992, 31-32).

As a solution, Collingwood seems to propose proceeding by comparison:

“5.47. When I ask myself this I get no answer. I can only say that feeling is more like being active than some experiences I could mention which are definitely experiences of being passive; but more like being passive than some which are definitely experiences of being active. 5.48. If I stuck to being honest [...] I should say: ‘I am aware of seeing the sky blue, and feeling the sunshine warm. I am not aware of feeling them actively as opposed to passively or passively as opposed to actively.’” (1992, 32-33).

Hence, the distinction is not operational, at least for the moment.^31^ If Collingwood’s approach is correct, then we should rather think that the question is either futile or can produce only an approximate answer.^32^

## Further Corollaries

If I am right and my argument is sound, the offered solution may prove useful for a broader project since it is a part of the nagging problem of how to build the general model of affectivity. For instance, the distinction between passivity and activity interweaves with distinctions of other categories applied to affectivity, such as bodily and mental. It would be interesting to see whether, to some extent at least, there are correspondences between the bodily and passivity and the mental and activity. Consider the following:

“3.4. [...] The pleasure of lying in a hot bath is called a bodily pleasure; the pleasure of reading Newton’s *Principia* is not. [...] 3.42. [...] in the case of the *Principia* the pleasure is the pleasure of thinking in certain ways. 3.43. If my pleasure in reading the *Principia* were derived from the actual look and smell and feel of the volume in my hands and under my nose and before my eyes I should call that, too, a bodily pleasure.” (Collingwood 1992, 15)

If one agrees with this, one will have to say that mental pleasure of reading the *Principia* is independent of this work’s (material) edition, the sensing and touching of which are bodily pleasures. The bodily pleasure of reading linked to “the actual look and smell [...] of the volume” may be produced by any volume of similar kind and not by the content of the volume, for the senses cannot apprehend the content. While thinking is commonly considered to be active and opposed to feeling, with mental pleasure we come to an active pleasure, since pleasure is produced, only or mainly, by means of thinking.

There is one feature, however, which may seem common to 1, 2b, and 3. I said that in group 1 the intention of *f-ing* may but does not have to be affective; in subgroup 2b being *f*-ed may but does not have to be affective; and in group 3 being *f*-ed may but does not have to follow *f*. In fact there are two differences between group 1 and subgroup 2b on the one hand and group 3 on the other, concerning their *may but does not have to.* 33 The first difference is that when in group 3 *f* doesn’t attain its object, *f* cannot considered to be a fully accomplished act of *f*-ing. This is because it matters whether *f* reaches its object or not. Even if acts belonging to group 3 may not be followed by affective responses, such responses should or are intended to follow. As such, they are not meant to remain in the agent but to pass over into the external object, i.e. the object of *f.* 34 It is different in group 1 and subgroup 2b: in group 1 the affective intention of *f* is separable from being *f*-ed and in group 2 being affectively *f*-ed does not result from *f*. There is no question of completion here. The second difference is that in group 3, what passes over into the object of *f*, if it does pass, and the result of being passed over into the object are both affective acts. In group 1 and subgroup 2b, the content of the causative act and the passive state, may be affective but this is not inherent to, respectively, being *f*-ed and *f*. And this is an important point. In group 3 the affective meaning of being *f*-ed is inseparable from the affective meaning of *f*. Being *f*-ed is the effect of *f* passing over into the object of *f* and, what is the same, the subject of feeling being *f*-ed. Otherwise, if feeling being *f*-ed were not be the effect of being *f*-ed, there would be nothing like being *f*-ed apart from a misleading way of speaking.

Let me end with the following observation which is to be taken as an illustration of how the distinction I suggest may be applied to the so-called basic feelings. For examples of basic feelings I draw on classical philosophers who were explicit about them, when explicitly treating the issue of affectivity. If we take basic feelings as they are listed by the Stoics, Aquinas, Descartes, and Spinoza and classify them in conformity with the division set above we obtain:

**Table:** 

	Aristotle^a^	Stoics^b^	Aquinas^c^	Descartes^d^	Spinoza^e^
(1) only passive	fear, anger	fear	fear, anger	–	–
(2) only active^f^	confidence/courage, joy, longing, shame, *aidos* ^g^	pleasure, pain	joy, sadness, courage, hope, despair	joy, sadness	pleasure, unpleasure
(3) both: passive and active	desire, envy, love/friendship, hatred, jealousy, pity	desire	love, hatred, desire, aversion	admiration, love, hatred, desire	desire

^a^In Aristotle they are not called basic. I form the list by combining the lists of examples he gives in *Ethica Nicomachea* 1105b: ἐπιθυμίαν ὀργὴν φόβον θάρσος φθόνον χαρὰν φιλίαν μῖσος πόθον ζῆλον ἔλεον, *Ethica Eudemia* 1220b: θυμὸν φόβον αἰδῶ ἐπιθυμίαν, and *Ars Rhetorica* 1378a20: [...] ὀργὴ ἔλεος φόβος [...]

^b^Four in the Stoics. See Diogenes Laertius VII, 111: εἶναι γένη τέτταρα, λύπην, φόβον, ἐπιθυμίαν, ἡδονήν

^c^Eleven in Thomas Aquinas, *ST* 1a2æ 23.4: “amor et odium, desiderium et fuga gaudium et tristitia [...] spes et desperatio, timor et audacia, et ira [...]”

^d^Six in R. Descartes, *Les passions de l’âme* [1649], art. 69: “admiration, amour, haine, désir, joie & tristesse”

^e^Three in Spinoza, *Ethica* [1677], *Prop. LIX*: “Omnes affectus ad cupiditatem, lætitiam vel tristitiam referuntur [...]”

^f^As for group 2 the examples I assign to it may still be divided into two subgroups, 2a (of which there is no intelligible passive voice) and 2b (of which the passive voice can be and is constructed but its sense is affectively irrelevant). Thus confidence/courage, joy, shame, pleasure, pain/unpleasure, joy, sadness, despair, and courage for group 2a, and *aidos*, longing and hope for group 2b

^g^This is difficult to translate (see *LSJ*: *reverence, awe, respect*). But given that the verb αἰδέομαι (*LSJ*: *to be ashamed*, *stand in awe of, fear*, *respect*) is a deponent form it cannot be passive. Other examples of deponent verbs relevant to affectivity are σέβομαι (*LSJ*: *feel awe* or *fear before God, feel shame*), γλίχομαι (*LSJ*: *cling to, strive after, long for*)

A remark is needed. It may well be that some assignments are uncertain. I acknowledge that desire may belong to group 2 rather than to group 3 (a similar doubt arises regarding longing, envy, jealousy and pity).^35^ This incertitude, however, does not speak against the division itself but rather speaks about an incorrect assignment of this or that element which, in turn, may result from an insufficient understanding of the nature of the element in question. Finally, if this assignment is correct a curious thing transpires: passive affective acts are represented least of all.^36^ And even more interesting is that two examples of passive affective acts, fear and anger, are the two emotions studied the most or referred to most often in current debate over emotions. That being so, it should not be surprising that seen in this light the class of emotions is generally treated as passive.

## Conclusion

In this paper I adopted Aquinas’ explanation of passivity and activity by means of an act remaining in the agent and an act passing over into external matter. I used it to set up a divide between immanent-type and transcendent-type acts. I then touched upon a grammatical distinction between three kinds of verbs (with only passive voice having affective meaning, with only active voice having affective meaning and with both passive and active voices having affective meaning). To argue for the activity and passivity of affectivity I exploited the last kind, this is, the group which includes acts of transcendent-type and yet has both voices, active and passive, possessing affective meaning. Although group 1 testifies to the passivity of affectivity and group 2 to its activity, by testifying to both group 3 is the best evidence of the activity and passivity of affectivity. It is unambiguous for it points to the same act being first active in the subject of *f*-ing and then mirrored in its object as being *f*-ed who, thereby, becomes the subject being *f*-ed. With this I believe we may say that affectivity as such is both active and passive, though in point of fact, every particular affective act belongs to one of three groups and may be either only passive or only active, or active mirrored by passive. This shows that affectivity is not homogeneous and should not be treated as uniform.

If so, this means that affectivity as a class is reducible neither to passivity nor activity. Moreover affectivity shares features with physical acts and with other classes of mental acts (e.g. thoughts), but without being identical to them. Hence it cannot be reduced to either physical acts or other mental acts. It should be therefore considered to be either a special, mental-like class of physical acts, or a special, physical-like class of mental acts, or a separate class of acts.^37^ The relation between acting subjects and objects acted-upon is not as strong as in the physical world, for there are cases of T-type acts uncompleted, that is, of acts in which loving deeply may not be reflected by being loved deeply (i.e. the feeling of being loved deeply), or even by being loved (i.e. the feeling of being loved) at all. On the other hand, loving deeply may be reflected in being loved deeply (i.e. the feeling of being loved deeply) and, most importantly, it is intended to be reflected by being loved deeply (i.e. the feeling of being loved deeply). Although the rarest, cases when affective *f*-ing is borne out by affective being *f*-ed represent the fullest or the most tangible dimension of affectivity. When active affectivity gives rise to passive affectivity, it resembles the physical world because activity and passivity concern the same particular affective episode.

## Footnotes

1 In what follows, I shall use *feeling* instead of *emotion*.

2 Of course all other things being equal and, importantly, with no change in subject between the two events that occur.

3 See e.g. Eisler (1910, 391): “Feeling is a subjective state in which the ego takes an immediate position in relation to modifications (suitable or not suitable) it encounters with regard to its lived experiences” (transl. J. Jansen).

4 The argument is put plainly by P. van Inwagen (1983, v - this is, as early as in the *Preface*): “If determinism is true, then our acts are the consequence of laws of nature and events in the remote past. But it’s not up to us what went on before we were born, and neither is it up to us what the laws of nature are. Therefore, the consequence of these things (including our present acts) are not up to us.” In my view, we cannot know whether determinism is true, since our knowledge, or rather what is supposed to be knowledge, would be then a result of the determinist chain and not of our reflecting, inquiring, arguing, providing proofs, and being convinced or not.

5 Think about Socrates’ following motive: “[...] we must not hearken to that captious argument: it would make us idle, and is pleasing only to the indolent ear, whereas the other makes us energetic and inquiring. [...] but that the belief in the duty of inquiring after what we do not know will make us better and braver and less helpless than the notion that there is not even a possibility of discovering what we do not know, nor any duty of inquiring after it - this is a point for which I am determined to do battle, so far as I am able, both in word and deed.” (Plato (1967c) 81d5-e1 & 86b7-c2, transl. W. R. M. Lamb)

6 For more see Zaborowski (2010).

7 Aristotle (1949) 1b27-2a4: “[...] or act or be acted upon [ἢ ποιεῖν ἢ πάσχειν] [...] act, for example to cut, to burn; to be acted upon, for example to be cut, to be burnt.”

8 Plato (1967a) 476b3-d1: [...] σκόπει δὴ καὶ τόδε: ἆρα εἴ τίς τι ποιεῖ, ἀνάγκη τι εἶναι καὶ πάσχον ὑπὸ τούτου τοῦ ποιοῦντος; [...] ἆρα τοῦτο πάσχον ὃ τὸ ποιοῦν ποιεῖ, καὶ τοιοῦτον οἷον ποιεῖ τὸ ποιοῦν; λέγω δὲ τὸ τοιόνδε: εἴ τις τύπτει, ἀνάγκη τι τύπτεσθαι; [...] καὶ εἰ σφόδρα τύπτει ἢ ταχὺ ὁ τύπτων, οὕτω καὶ τὸ τυπτόμενον τύπτεσθαι; [...] τοιοῦτον ἄρα πάθος τῷ τυπτομένῳ ἐστὶν οἷον ἂν τὸ τύπτον ποιῇ; [...] οὐκοῦν καὶ εἰ κάει τις, ἀνάγκη τι κάεσθαι; [...] οὐκοῦν καὶ εἰ τέμνει τι, ὁ αὐτὸς λόγος; τέμνεται γάρ τι. [...] καὶ εἰ μέγα γε ἢ βαθὺ τὸ τμῆμα ἢ ἀλγεινόν, τοιοῦτον τμῆμα τέμνεται τὸ τεμνόμενον οἷον τὸ τέμνον τέμνει;

9 Thomas Aquinas (1947), *ST* 1a2æ 26.2: “passio est. effectus agentis in patiente”. There is another one, in which Aquinas focuses on motion rather than on being effect: “The movements of the sensitive appetite, are properly called passions, as stated above (Q[22], A[3]). Now every emotion arising from a sensitive apprehension, is a movement of the sensitive appetite” (transl. The Fathers of the English Dominican Province, *ST* 1a2æ 31.1: “motus appetitus sensitivi proprie passio nominatur, sicut supra dictum est. Affectio autem quaecumque ex apprehensione sensitiva procedens, est. motus appetitus sensitivi.”)

10 Descartes (1901), p. 310, vv. 16–21: “[...] on peut generalement nommer passions toutes les pensées qui sont ainsy excitées en l’ame sans le concours de la volonté, & par consequent, sans aucune action qui viene d’elle, par les seules impressions qui font dans le cerueau, car tout ce qui n’est point action est passion.”

11 Descartes (1901), p. 311, vv. 8–13: “[...] lorsqu’elle vse de sa volonté pour se determiner a quelque pensée qui < n’est pas seulement intelligible, mais imaginable, cette pensée > fait vne nouuelle impression dans le cerueau, cela n’est. pas en elle vne passion, mais vne action, qui se nomme proprement imagination.”

12 For an analysis of a similar case borrowed from mental realm. See Plato (1969) 430e11-431a6: ““Now the phrase ‘master of himself’ is an absurdity, is it not? For he who is master of himself would also be subject to himself, and he who is subject to himself would be master. For the same person is spoken of in all these expressions.” “Of course.” “But,” said I, “the intended meaning of this way of speaking appears to me to be that the soul of a man within him has a better part and a worse part, and the expression self-mastery means the control of the worse by the naturally better part. [...]” (transl. P. Shorey)

13 It could be added that this also happens without his intention, deliberation, will, etc. - but these are still different kinds of concepts that are not needed at this stage of the distinction.

14 After Kenny (1963, 196). Aquinas wording is: “action transit in exteriorem materiam” and “manet in agente”.

15 I leave aside the case of a look - or rather of a gaze - and similar that affects the object.

16 No modification in the object, for there is one in the subject.

17 See Gordon (1986, 373): “I begin the argument of this paper with a datum on which consensus is easily attained: the *grammatical* passivity of emotion descriptions. [...] Grammatical evidence suggests that the “passivity” of states such as fear, anger, and jealousy is not an invention of philosophers or psychologists.” I think neither *consensus* nor *easily* occurs.

18 It could be remarked that in some cases the meaning of the passive voice is not to be taken literally and can be replaced with a noun or an adjective, e.g. *I am frightened* with *I have a fright*. This happens when no particular cause of being frightened/having a fright can be indicated. See also Johnson-Laird & Oatley (1989, 100–102).

19 Gordon himself gives two such examples (one adjective, *sorry*, and one verb, *enjoy*) that do not “bear historical traces of transitivity”. Gordon doesn’t spell out what to do with them. By speaking about “historical traces” Gordon, in my view, relies on the way people have been thinking about affectivity rather than on the ontic structure of affectivity.

20 One may remark that if I am rejoicing, an external observer can be affected by my rejoicing - infected, so to speak, by my expression of that joy. But this is again what may but does not have to happen and, as such, can be put aside here.

21 By *absent* I mean here *not present in any way whatsoever*. At the risk of circularity I would say that when the object of *f* is not present in any way whatsoever, it cannot be that an object is passing over into it and a modification cannot be instantiated. But what about love and other elements of group 3? Does love occur only in the presence of the beloved? And if not, what is the difference between loving and missing? May the beloved feel being loved when the lover is absent? I am not able to conceptualise this here.

22 Verbs of group 2b remind us of *thinking about* (see above), which may pass over into the object of thinking about only if additional conditions are fulfilled. So here: I may feel (or only: I may know because I have learnt) that I am missed but I don’t feel (or know) this simply because of someone’s missing me. The additional conditions are not provided by the very act of missing per se. But this generates further question: if I feel (or know) that I am missed only if I have learnt so, say I have been told so, how can I know if my feeling is correct or my knowledge accurate, i.e. if the content of feeling or knowledge corresponds the state of affairs?

23 See Plato (1955) 212c4: ὁ μὲν φιλεῖ, ὁ δὲ φιλεῖται (transl. W. R. M. Lamb: “the one loves and the other is loved”) and also 212c1-d5: ἀντιφιλεῖσθαι [...] ἀντιφιλοῦν (“being loved reciprocally [...] loving reciprocally”).

24 See Plato (1969) 417b2: μισοῦντες δὲ δὴ καὶ μισούμενοι (“hating and being hated”), Plato (1967b) 697d5-6: ἐχθρῶς τε καὶ ἀνηλεήτως μισοῦντες μισοῦνται (transl. R. G. Bury: “thus they both hate and are hated with a fierce and ruthless hatred”).

25 To simplify, here and throughout this paper, I consider verbs *f* with their objects as alive persons, for I admit that a non-living object of *f* is unable to feel that it is *f*-ed.

26 I don’t consider a case when someone may feel loved without being loved, that is, without an act of loving. I would take this to be an example of affective hallucination.

27 For obvious reason loving what-is-not-a-person (animals, plants, and other living organisms as well groups, nations, etc.) lies beyond the scope of the paper.

28 Here it becomes clear that *being loved* makes sense only if it stands for *feeling being loved*. Symmetrically, we could say *feeling loving*, but we don’t say this because to say *loving* is enough.

29 The same is valid for other elements of this group, e.g. admiring a person versus the person being admired, even though here the intention of acting upon the admired person seems less obvious than in the case of love. But to see it, please imagine that you admire someone and keep your admiration inside, that is, you keep your admiration hidden from the person admired so that she is unable to spot it. Here a distinction between admiring and contemplation (which is for the sake of itself) may be useful.

30 To look for conditions on which feeling being *f*-ed does follow *f* would mean embarking on a new issue: the authenticity of T-type affectivity.

31 See Collingwood (1992, 33): “5.49. [...] for the distinction between activity and passivity involves the distinction between oneself and what is not oneself, and this distinction, as I shall explain later on (chapter viii), is first made at a far more highly developed stage of mental development than that which we are now exploring.’”

32 For more see Zaborowski (2016).

33 Compare A. Kenny (1963, 196): “if Peter has looked at his house, it may now be exactly the same as it was before he looked at it” - what does “it may” mean? *May* it be different because of Peter’s looking at it? One may wonder if it *may not* be exactly the same, that it *may* change because of Peter’s looking at it and if so how this occurs. Does Kenny mean that looking may modify the house as painting does? If it *may* then where is the difference between painting and looking, physical and mental action? Is the difference between *passing over into external matter* and *remaining in the agent* then essential or accidental?

34 A different approach in Waterlow (1970, 92) who puts *to love* and *to miss* in the same group of non-affective verbs.

35 The case of desire is complex: if it is understood as desiring an object qua present physically or otherwise it belongs to this group. If however the object is understood qua absent then it belongs to group 2b (see above). See Plato, *Phaedrus* 255d6–8: καὶ ὅταν μὲν ἐκεῖνος παρῇ, λήγει κατὰ ταὐτὰ ἐκείνῳ τῆς ὀδύνης, ὅταν δὲ ἀπῇ, κατὰ ταὐτὰ αὖ ποθεῖ καὶ ποθεῖται (“when he [the lover] is present like him [the beloved] stops his pain, but when he is absent like him he longs for him and is longed for by him”). So I am in trouble: either ποθεῖν belongs to 2b and ποθεῖται describes the beloved’s position, or it belongs to 2b and the absence is not total, so that the beloved *may* feel being missed and *actually* feels that he is missed or, otherwise, my interpretation is wrong. According to *LSJ* ποθεῶ *= long for, yearn after* with a qualification: *what is absent*. But if so, my distinction between 2b and 3 fails. If the absence of the object is total, the act of longing *remains in the agent*. But how then to conceive of the passive voice? Imagine you are asked to say what you feel when you are longed for or missed (to use a direct object), but to avoid merging it with your missing him who misses you.

36 This flies in the face of Gordon’s claim about “great majority of passive emotions”.

37 By means of digression let me say that affectivity often lies in the middle, between two opposed terms. E.g. the same works for direction of fit: do feelings have mind-to-world (as beliefs) or world-to-mind (as desires) fit? Feelings as a class seem not to belong to either. This question has recently been analysed by Yang (2016, 39–41): “A fear is inappropriate if the mind doesn’t fit the world, and a hope is wrong if the world doesn’t fit the mind. For some emotions, we strive to bring about their objects and, for others, we try to avoid their objects. [...] We could say that pride sometimes involves mind-to-world (belief), sometimes world-to-mind (desire), or sometimes neither directions of fit. It follows that it is difficult to say that emotions have either direction of fit.”
